# Effect of Yttrium and Rhenium Ion Implantation on the Performance of Nitride Ceramic Cutting Tools

**DOI:** 10.3390/ma13204687

**Published:** 2020-10-21

**Authors:** Dmitrij Morozow, Zbigniew Siemiątkowski, Edwin Gevorkyan, Mirosław Rucki, Jonas Matijošius, Artūras Kilikevičius, Jacek Caban, Zbigniew Krzysiak

**Affiliations:** 1 Faculty of Mechanical Engineering, Kazimierz Pulaski University of Technology and Humanities in Radom, ul. Stasieckiego 54, 26-600 Radom, Poland; d.morozow@uthrad.pl (D.M.); z.siemiatkowski@uthrad.pl (Z.S.); 2 Department of Quality, Standardization, Certification and Manufacturing Technology, Ukraine State University of Railway Transport, 7 Feuerbach sq., 61010 Kharkiv, Ukraine; cermet-u@mail.com; 3 Institute of Mechanical Science, Vilnius Gediminas Technical University, J. Basanavičiaus str. 28, LT-03224 Vilnius, Lithuania; jonas.matijosius@vgtu.lt (J.M.); arturas.kilikevicius@vgtu.lt (A.K.); 4 Faculty of Mechanical Engineering, Lublin University of Technology, Nadbystrzycka 36, 20-618 Lublin, Poland; j.caban@pollub.pl; 5 Faculty of Production Engineering, University of Life Sciences in Lublin, Głęboka 28, 20-612 Lublin, Poland

**Keywords:** cutting, turning, titanium alloy, tool performance, ion implantation, edge wear

## Abstract

In the paper, the results of experimental investigations of ion implanted cutting tools performance are presented. The tools, made out of Si_3_N_4_ with additives typically used for turning of Ti-6Al-4V alloy, underwent implantation with ions of yttrium (Y^+^) and rhenium (Re^+^) using the metal vapor vacuum arc method. Distribution of ions on the tool surface was measured. The cutting tools were tested in turning process with measurement of cutting forces and analysis of wear. A rather unexpected result was the increased wear of the tool after Y^+^ implantation with 1 × 10^17^ ion/cm^2^. It was demonstrated, however, that the tool after Y^+^ 2 × 10^17^ ion/cm^2^ ion implantation provided the best machining performance.

## 1. Introduction

Due to its extraordinary properties, titanium and titanium alloy Ti-6Al-4V is widely used in various branches of industry [1,2,3], such as aircraft, automotive industry and optoelectronics [4,5], or medicine [6]. In the field of orthopedics and in dental prosthetics, titanium found a wide range of applications [6,7]. Its high corrosion resistance is attributed to passive layers consisting of amorphous TiO_2_, formed on the surfaces [8,9,10]. It may be characterized as a very difficult-to-cut material due to its endurance and relative elongation (6–15%). Moreover, low heat transfer ability causes that the thermal energy does not dissipate easily by conduction, which results in increase of temperatures in the cutting contact area [11]. A similar situation takes place in the case of cobalt alloys [12]. As a consequence, large amounts of heat are not carried away with the chipped material, but penetrate into the cutting tool [13], which require application of new cooling methods [14]. In the research on Ti-6Al-4V alloy machining, special attention is paid to the additive manufactured details cut with solid ceramic tools [15].

It is widely acknowledged that ceramic tools have good thermal stability and perform very well during high temperature cutting processes [16]. Nevertheless, the inserts designed to cut the difficult-to-machine materials are coated with special coatings. These are typically multilayer compositions, with outer layers reducing friction coefficient and inner layers protecting the tool from the heat generated at the tool–chip interface [17].

Specific microstructure in the coating, and especially in its subsurface layers, and thus enhanced hardness and a desirable residual stress can be achieved using PVD (physical vapor deposition) methods [18] or with ion implantation techniques, which is known to be suitable for ceramics [19]. Almost all characteristics of the tool material increasing its wear resistance were found to be changed after ion implantation [20]. It was reported that the tools PVD-coated with (Ti, Al)N + TiN had the longest tool life and were recommended for face milling of titanium alloys [21]. The process of deposition can be supported with IBAD (ion beam assisted deposition), to induce changes in hardness or to generate desirable residual stresses in the subsurface layer [22]. This way, substantial improvement of the cutting tools performance and reduction of their wear can be reached. Wear conditions of different cutting tools could increase the cost of production and cause substantial effect on carbon emissions [23]. There are many factors affecting the wear of cutting tools, such as vibration [24] and cutting forces [25]. There are publications on micro end milling of Ti-6Al-4V that focused on wear behavior and performance of tungsten carbide cutting insert with TiAlN coating [26]. However, there are very few reports about the ion implantation impact on the lifetime of ceramic inserts at increased temperatures in industrial conditions [27]. The present work is dedicated to the influence of yttrium (Y^+^) and rhenium (Re^+^) ions implantation into the rake face of nitride ceramics cutting inserts on their performance in real working conditions.

## 2. Materials and Methods 

The research was focused on the cutting tools performance during machining of a typical α+β dual-phase titanium alloy Ti-6Al-4V. Its microstructure consists of hexagonal close packed (HCP) α and body centered cubic (BCC) β phases [28]. Since α phase normally precipitates from β phase, the arrangement of phases is determined by the heat treatment conditions [29]. Physically, α phase is stabilized by aluminum, while β phase by vanadium, so that the aluminum addition provides an increase of strength, while the vanadium addition allows maintaining plasticity [30]. Values of the Young modulus can have quite a large scattering, reportedly varying from 94 to 118 GPa [31]. Chemical composition of the alloy is the following: Al—6.5%, V—4.3%, Fe—0.18%, C—0.01%, N—0.13%, and O—0.13% by weight [32].

Thermal conductivity of titanium alloys is low, ca. 1/6 that for steel [33], and it can be assumed as 6.7 W/(m·K) [34]. It results in heat concentration on the cutting edge and the rake face of the tool during machining. The temperature in the chip–tool interface when turning Ti-6Al-4V can reach 1200 °C [35]. The material strengthening phenomena takes place, and, as a consequence, the tool wear is increased, friction and cutting forces rise up, and the cutting edge is quickly damaged. According to the available reports, hardness of annealed Ti-6Al-4V with alpha-beta microstructure is ca. 350 HV [33].

**Figure 1:**
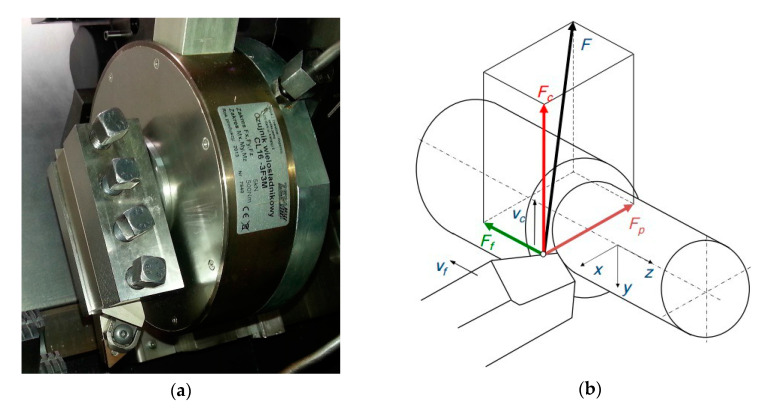
CL16-3F3M device: (**a**) The overall view; (**b**) scheme of the measured forces components.

For the turning tests, the samples made out of Ti-6Al-4V alloy with diameter ∅60 mm and length *l* = 35 mm. The tests of cutting were performed using the machining center DMG NEF400 CNC (Bielefeld, Germany). The cutting forces were measured with the dynamometer CL 16-3F-3M made by ZEPWN (Marki, Poland) shown in Figure 1. It was able to measure cutting forces during machining process, and its uncertainty was assumed below 0.45% of the actually measured value [17]. The sampling was performed each 0.1 s.

There are some recommendations on the machining parameters for different titanium alloys [36]. In general, the highest cutting speeds *v_c_* between 95 and 210 m/min can be applied to non-alloyed titanium, but an increase of vanadium and chromium content with a harder β phase requires smaller cutting speeds. For the dual-phase alloy Ti-6Al-4V, the recommended cutting speed was 48–100 m/min, and in experiments, it was *v_c_* = 50 m/min at feed rate *f* = 0.15 mm/rev and cutting depth *a_p_* = 0.5 mm.

Since Si_3_N_4_-based ceramics appeared to be superior to Al_2_O_3_-based ones as the material of cutting tools [37], the former was chosen for the experiments. Cutting tools were used with inserts TNGA 160408E (IS9) made out of nitride ceramics IS9 (Si_3_N_4_ with additives), fixed in holders MTJNL 2020K-16W-M produced by ISCAR company (Tel Aviv, Israel). The geometry of cutting tools was as follows: α = 6°; γ = −6°; *κ_r_* = 93°; *r_ε_* = 0.8 mm. No coolants were applied.

Before the turning tests, some of the applied inserts underwent the ion implantation procedure. The rake surfaces of the cutting inserts underwent ion implantation with the respective ions of yttrium (Y^+^) and rhenium (Re^+^) in order to modify the properties of the outer layer. For that purpose, the device TITAN (HCEI, Tomsk, Russia), equipped with a spark source of metal vapor MEVVA type (metal vapor vacuum arc) was used. Since tribological aspects play important role in the tool wear [38], the parameters of the ion implantation process were chosen according to the tribological research described in [39]. Table 1 presents applied ion doses and beam energy of the process.

The implantation procedure was performed at the Polish National Center of Nuclear Research (NCBJ, Świerk, Poland). Its results were assessed with a scanning electron microscope EVO MA10 produced by ZEISS (Jena, Germany).

## 3. Results and Discussion

The experiments were aimed on assessment of the performance of ion implanted cutting tools compared to the typical unimplanted ones. To reach that goal, three directions were explored, namely, quality of the ion implantation, cutting forces during machining, and tool wear. The results are presented and discussed in the respective subsections.

### 3.1. Ions Distribution on the Rake Surface

**Figure 2:**
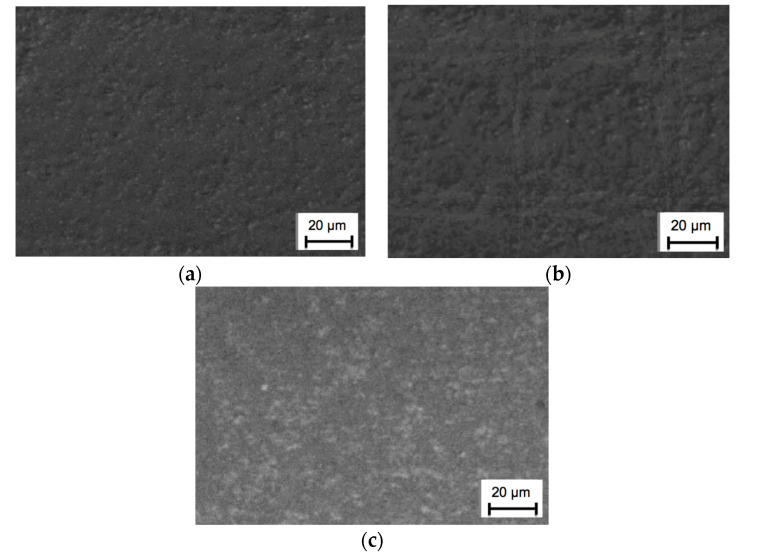
Analyzed surfaces: (**a**) Unimplanted insert surface; (**b**) Surface after yttrium ion implantation (dose 2 × 10^17^ ion/cm^2^); (**c**) Surface after rhenium ion implantation (dose 2 × 10^17^ ion/cm^2^).

**Figure 3:**
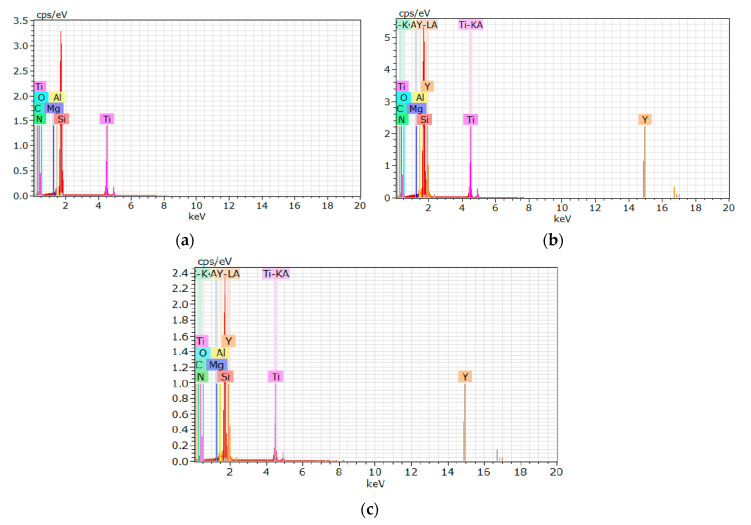
EDS analysis of different chemical compositions of the IS9 inserts: (**a**) Before ion implantation; (**b**) after yttrium ion implantation at dose 1 × 10^17^ ion/cm^2^; (**c**) after yttrium ion implantation at dose 2 × 10^17^ ion/cm^2^.

Rake surfaces of nitride ceramic inserts IS9 before and after ion implantation can be seen in Figure 2. It is noteworthy that the general appearance of the surface did not change, because of a very thin coating of ions. Figure 3 presents the results obtained from the energy dispersive spectroscopy (EDS) measurement.

**Figure 4:**
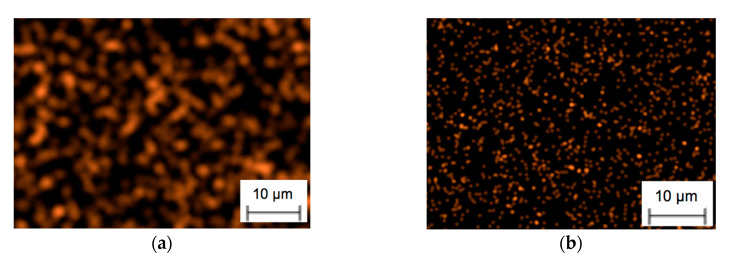
Yttrium (**a**) and rhenium (**b**) distribution on the nitride ceramic surface after ion implantation.

It can be seen that the chemical composition after yttrium ion implantation at both doses contains some amounts of Y, absent in the original material. To assess distribution of the material on the rake surface, the maps were prepared with the points of higher density and brightness on the areas with higher substance content. There was no significant difference in mappings representing elements after ion implantation at dose 1 × 10^17^ ion/cm^2^ or 2 × 10^17^ ion/cm^2^. An example of such a map is shown in Figure 4, where steady distribution of both yttrium and rhenium is clearly seen.

Lower brightness may suggest that Re^+^ ions adhered to the nitride ceramic surface in smaller degree than ions Y^+^. Nevertheless, presence and steady distribution of the ions that made up a coating, is clear and obvious, which confirms high quality and surface integrity of the investigated cutting tools.

### 3.2. Cutting Forces Measurement

**Figure 5:**
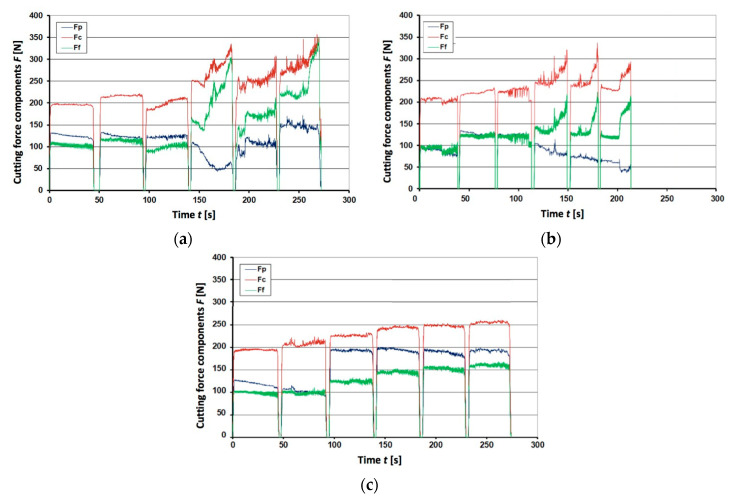
Registered force components *F_p_*, *F_c_*, and *F_f_* for the insert IS9 without implanted ions: (**a**) 1st edge; (**b**) 2nd edge; (**c**) 3rd edge.

**Figure 6:**
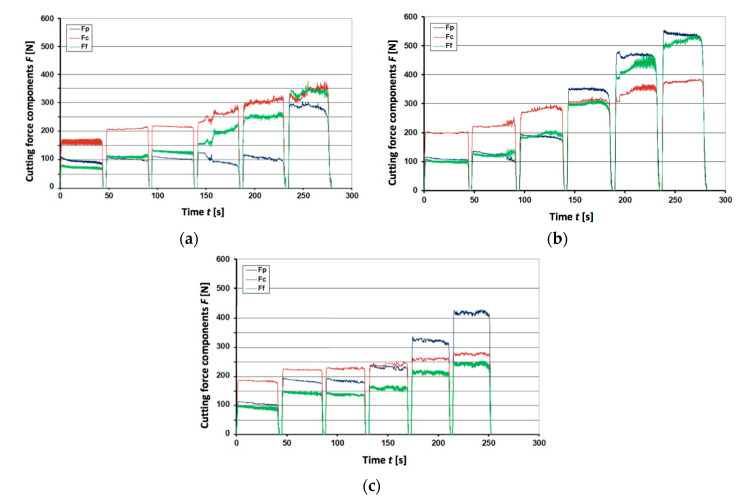
Registered force components *Fp*, *Fc*, and *Ff* for the Re^+^ implanted at dose 2 × 10^17^ ion/cm^2^ insert IS9: (**a**) 1st edge; (**b**) 2nd edge; (**c**) 3rd edge.

**Figure 7:**
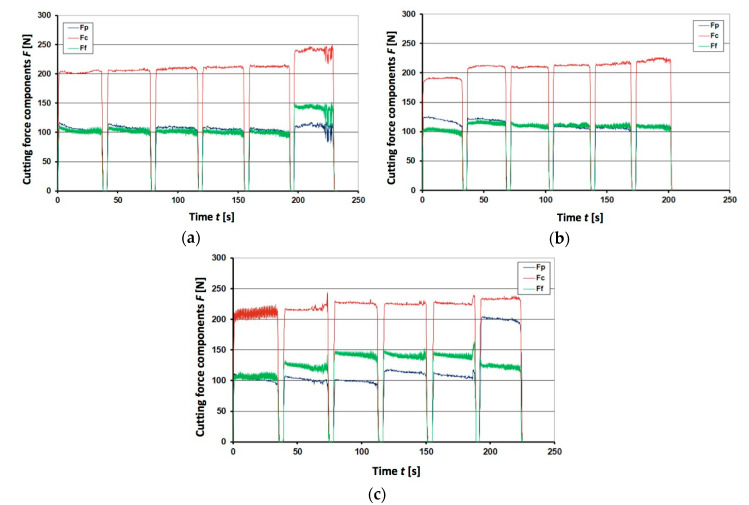
Registered force components *F_p_*, *F_c_*, and *F_f_* for the Y^+^ implanted at dose 2 × 10^17^ ion/cm^2^ insert IS9: (**a**) 1st edge; (**b**) 2nd edge; (**c**) 3rd edge.

The cutting forces during tests are represented by graphs shown in Figure 5, Figure 6 and Figure 7. They were plotted for the unimplanted cutting inserts, rhenium (Re^+^) implanted and yttrium (Y^+^) implanted ones, respectively. Since the obtained results appeared very different for three edges of each triangular inserts, the respective plots for each edge are shown separately. The plots represent the resulting machining force through its three components, namely, cutting force *F_c_*, feed force *F_f_*, and passive force *F_p_*. The graphs contain 6 cycles of interrupted cutting each, since the tool life tends to be reduced in the interrupted cutting method due to the high impact loads during the entry and the exit from the cut [40].

From Figure 5, it is seen that 1st and 2nd edges were damaged after ca. 100 s of work, so that further cutting did not provide required quality. In case of the 3rd edge, damage was rather steady and the edge was blunting without larger cracks and chipping, though unstable cutting force *F_c_* between 50 and 100 s might indicate some kind of accelerated wear or microdamage. Looking at the first three cycles in Figure 6, it is difficult to find substantial improvement in case of rhenium ion implantation. Further cycles of the Re^+^ implanted insert appear more stable, though perform higher forces, which is not the case for the 3rd edge of unimplanted insert, however. In addition, passive force *F_p_* is clearly increasing from 100 up to 400 and even 500 N for two edges of the Re^+^ implanted insert. Steady twofold and even 5-fold increase of feed force *F_f_* is noteworthy: it takes place in all edges of the Re^+^ implanted insert in a similar way. In this respect, the tools implanted with yttrium ions appear to be much more advantageous because of stable performance during 200 s in all 6 cycles, as it is seen in Figure 7. Detailed analysis of the graphs obtained for Y^+^ implanted tools at dose 1 × 10^17^ ion/cm^2^ (not shown here) proved that after 3–4 cycles, when the layer of yttrium was worn out, forces increased and lost stability.

Special attention should be paid to the passive force component *F_p_*, responsible for penetration into the machined material. When the edge loses its sharpness, larger *F_p_* is required for overcoming the material’s resistance. In the graphs for unimplanted inserts, its value is rather unstable, varying between 50 and 150 N (Figure 5a,b). Its jump from 100 to 200 N after 100 s in Figure 5c may be attributed to some sort of chipping damage of the cutting edge. A similar sharp increase of the passive force component *F_p_* is seen in Figure 6a, but after 230 s of working and reaching higher values of almost 300 N. Other rhenium-implanted edges performed gradual increase of *F_p_* after each interruption. In this respect, the yttrium implanted inserts performed exceptionally well, keeping a very steady *F_p_* value of ca. 100 N with some vibrations after 230 s (Figure 7a) or jump up to 200 N after 200 s (Figure 7c).

**Figure 8:**
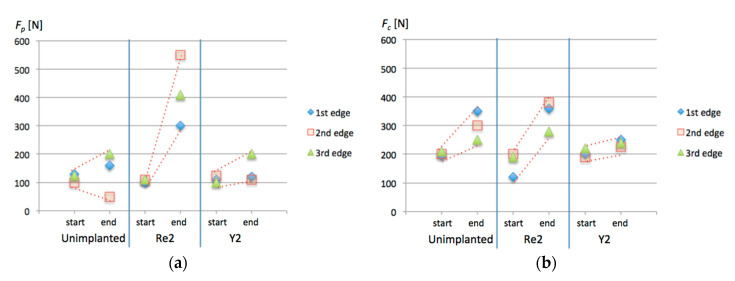

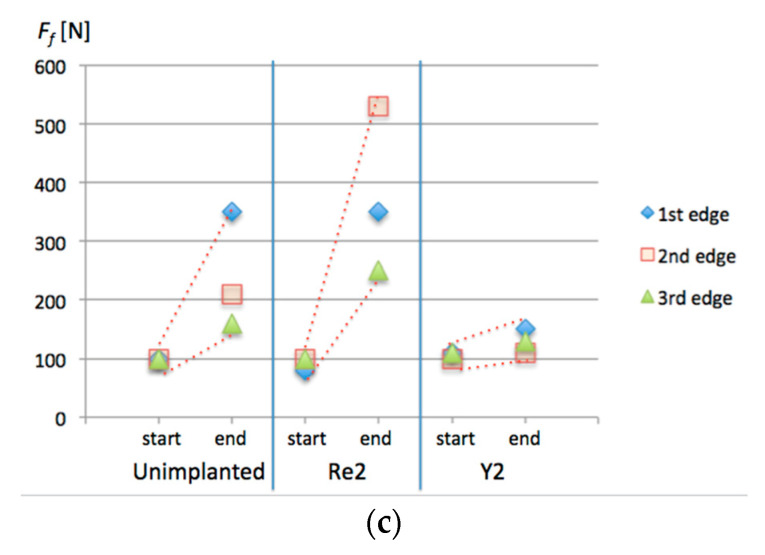
Registered force components: (**a**) *F_p_*; (**b**) *F_c_*; (**c**) *F_f_*. The respective symbols along X-axis denote start and end points of turning tests for cutting inserts: Unimplanted; Re2—Re^+^ (2 × 10^17^ ion/cm^2^) implanted edges: Y2—Y^+^ (2 × 10^17^ ion/cm^2^) implanted edges.

In order to provide more generalized insight, the initial and final values of forces in each turning test were gathered in single graphs presented in Figure 8. Dashed lines were added to emphasize the general trend for each cutting tool. It should be noted that for yttrium ion implanted surfaces, dispersion of all forces components values in the end points is the smallest.

### 3.3. Wear of the Cutting Tool

**Figure 9:**
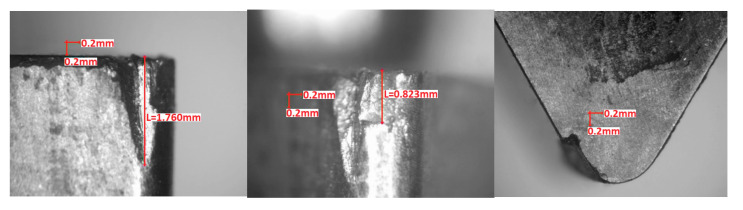
Unimplanted TNGA 160408E (IS9) edge after turning test (*VB_N_* = 1.8 mm, *VB_C_* = 0.8 mm).

**Figure 10:**
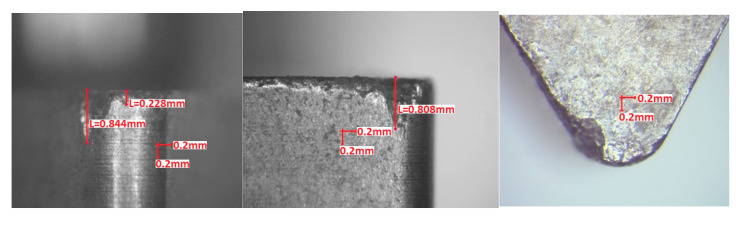
Rhenium implanted at dose 2 × 10^17^ ion/cm^2^ TNGA 160408E (IS9) edge after turning test (*VB_N_* = 0.8 mm, *VB_C_* = 0.2 mm).

**Figure 11:**
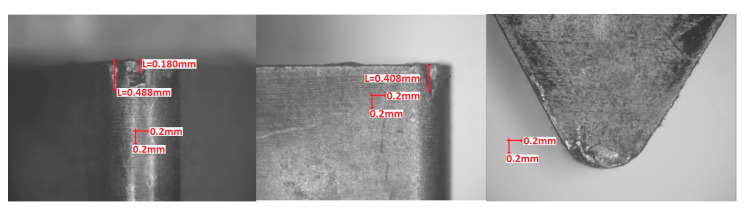
Yttrium implanted at dose 2 × 10^17^ ion/cm^2^ TNGA 160408E (IS9) edge after turning test (*VB_N_* = 0.4 mm, *VB_C_* = 0.2 mm).

**Figure 12:**
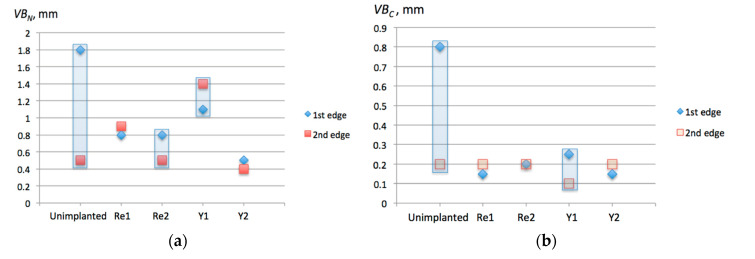
Wear parameters of TNGA 160408E (IS9) inserts measured on two edges each: (**a**) *VB_N_*; (**b**) *VB_C_*. The respective symbols along X-axis denote: Unimplanted 1st and 2nd edge; Re1—Re^+^ (1 × 10^17^ ion/cm^2^) implanted edges; Re2—Re^+^ (2 × 10^17^ ion/cm^2^) implanted edges: Y1—Y^+^ (1 × 10^17^ ion/cm^2^) implanted edges; Y2—Y^+^ (2 × 10^17^ ion/cm^2^) implanted edges.

Visual inspection of the cutting edges and rake faces after turning tests was in conformity with the observations made from forces graphs. However, in order to make the results more objective, wear parameters *VB_N_* (notch wear, mm) and *VB_C_* (tool corner wear, or flank wear of tool corner, mm) were calculated according to the Standard PN-ISO 3685:1996. Figure 9, Figure 10 and Figure 11 present examples of the worn edges photomicrographs of the unimplanted, rhenium (Re^+^) implanted, and yttrium (Y^+^) implanted inserts, respectively. In Figure 12, values of the obtained wear parameters are collected in the bar diagram. 

From Figure 12, where evident prevalence of implanted tools is seen, no clear trend can be derived, though. Differences of *VB_N_* values between two edges of the same insert sometimes appear very large, especially in case of the insert without ion implantation. One would expect smaller wear for Re^+^ 2 × 10^17^ ion/cm^2^ than for Re^+^ 1 × 10^17^ ion/cm^2^, which did not take place in case of the 1st edge. In addition, rather unexpected was the increased wear of for Y^+^ 1 × 10^17^ ion/cm^2^ (Y1). Nevertheless, the column Y2 corresponding with Y^+^ 2 × 10^17^ ion/cm^2^ insert stays in agreement with data obtained from cutting forces measurement. Namely, the tool after Y^+^ 2 × 10^17^ ion/cm^2^ ion implantation provided the best machining performance.

The *VB_C_* values in Figure 12b did not reveal any distinguishable trend. Again, the tool after Y^+^ 2 × 10^17^ ion/cm^2^ ion implantation (Y2) was worn in the smallest degree, even though similar results were obtained for Re^+^ 1 × 10^17^ ion/cm^2^ (Re1). In any case, however, *VB_C_* values for ion implanted surfaces were in range between 0.10 and 0.25, which was less than 1/3 of the respective *VB_C_* = 0.8 for the unimplanted tool.

Thus, in terms of tool wear decrease, ion implantation with larger doses did not provide evident improvement than that of smaller doses, while compared to the unimplanted tools, the implanted ones they performed substantially better. *VB_N_* values for implanted tools were at least 20% and at best 75% lower.

## 4. Conclusions

The research provided important initial qualitative information on impact of ion implantation on performance of cutting tools with difficult-to-machine materials. Wear analysis and turning tests of inserts TNGA 160408E made out of silicon nitride ceramics IS9 (Si_3_N_4_ with additives), after ion implantation with rhenium and yttrium, enabled formulating the following conclusions:

- implantation of ions to the rake face of ceramic cutting tools is the effective way to improve their properties;
- in particular, implantation of yttrium ions significantly decreased cutting resistance during dry machining of titanium alloy Ti-6Al-4V, compared to the inserts without ion implantation as well as to the ones implanted with rhenium ions;
- wear of cutting edges after ion implantation was smaller than unimplanted ones, thus providing longer lifetime and better performance of tools with ion implanted surfaces;
- significant improvement was obtained only with higher doses of ions, namely, Y^+^ 2 × 10^17^ ion/cm^2^;
- in further research, more attention will be paid to the cutting tools implanted with yttrium ions in order to collect in-depth quantitative results.

The proposed methodology can be used in order to improve performance and to prolong the lifetime of the ceramic cutting tools applied for machining of the titanium alloys, as well as other difficult-to-cut materials. Further investigations will enable assessment of the ion implantation effect on performance of newly developed nanocomposite cutting tools.

## Figures and Tables

**Table 1:** Ion implantation parameters.

No.	Insert	Ion Type	Ion Dose (ion/cm^2^)	Beam Energy (keV)
1	TNGA 160408E (IS9, Si_3_N_4_-based ceramic)	Re^+^	1 × 10^17^ 2 × 10^17^	65
2	TNGA 160408E (IS9, Si_3_N_4_-based ceramic)	Y^+^	1 × 10^17^ 2 × 10^17^	65
